# Universal Tobacco Screening and Opt-Out Treatment Referral Strategy Among Patients Diagnosed With Cancer by Race and Ethnicity

**DOI:** 10.1001/jamanetworkopen.2024.9525

**Published:** 2024-04-22

**Authors:** Gleneara E. Bates-Pappas, Elizabeth Schofield, Lou-Anne R. Chichester, Chris Kotsen, Lisa Carter-Bawa, Graham W. Warren, Jamie S. Ostroff

**Affiliations:** 1 Department of Psychiatry and Behavioral Sciences, Memorial Sloan Kettering Cancer Center, New York, New York; 2 Center for Discovery and Innovation, Hackensack Meridian Health, Hackensack, New Jersey; 3 Georgetown Lombardi Comprehensive Cancer Center, Washington, DC; 4 Department of Radiation Oncology, Medical University of South Carolina, Charleston, South Carolina

## Abstract

This quality improvement study examines whether a universal screening and opt-out referral model could promote racial and ethnic equity in access and use of tobacco treatment among patients with cancer.

## Introduction

Persistent smoking is associated with adverse clinical outcomes in cancer care.^1^ Black and Hispanic patients with cancer typically have lower access to and use of tobacco treatment services compared with White patients.^1^ We examined whether a universal tobacco screening and opt-out tobacco treatment referral model could promote equity in access and use of tobacco treatment among patients with cancer.^1^

## Methods

This quality improvement study was deemed exempt by the Memorial Sloan Kettering Cancer Center (MSKCC) institutional review board as a retrospective research study and followed the SQUIRE reporting guideline.

We analyzed data from patients diagnosed with cancer at MSKCC between January 1, 2018, and December 31, 2022. In 2011, MSKCC implemented universal screening of tobacco use and adopted an opt-out tobacco treatment referral as standard of care (eFigure in Supplement 1). Patients identified as currently smoking were offered tobacco treatment. Treatment acceptance was defined as scheduling at least 1 session. Patients not currently using tobacco were classified as ineligible.

We analyzed differences in tobacco use, tobacco treatment referral, and acceptance by race and ethnicity using χ^2^ and *t* tests. Race and ethnicity were self-reported, and the categories were derived from the electronic health record. Data were analyzed using R, version 4.3.1 (R Foundation). The significance threshold was a 2-sided *P* < .05.

## Results

As shown in the Figure and Table, among 302 971 patients seen during the study period (58.7% female and 41.3% male; mean [SD] age, 61.9 [14.6] years), the prevalence of current tobacco use was 6.1% and varied significantly by race, with Black or African American patients reporting the highest tobacco use (7.1% compared with 3.8%, 6.2%, and 6.1% for patients of Asian, White, and other race, respectively; *P* < .001). There were no observed differences in tobacco prevalence between Hispanic or Latino/e/a/x and non-Hispanic or Latino/e/a/x patients; however, prevalence was lower among patients with missing ethnicity data. Of 18 475 patients identified as currently using tobacco, 87.1% were referred for tobacco treatment. Of those referred, 87.6% were eligible, of whom 69.3% were reached for scheduling. Overall acceptance of tobacco treatment among patients reached was 54.8% and was highest among Black or African American patients (66.3% compared with 46.5%, 53.7%, and 58.1% for Asian, White, and other races, respectively; *P* < .001) and Hispanic or Latino/e/a/x patients (60.5% compared with 54.3% for non-Hispanic or Latino/e/a/x patients; *P* = .005).

**Figure.**
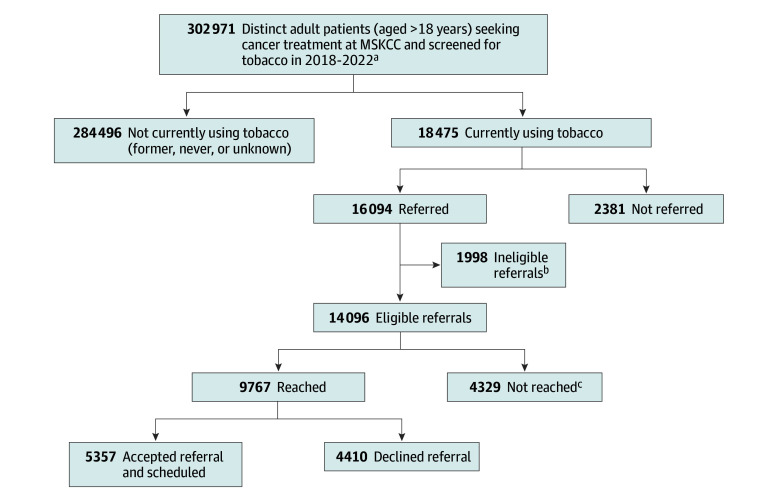
Universal Tobacco Assessment and Referral Clinical Workflow ^a^Excluded patients who did not receive cancer treatment at Memorial Sloan Kettering Cancer Center (MSKCC) (ie, consults only and second opinion only). ^b^For this analysis, patients who reported using cannabis only or who had a tobacco use status of never or former at the time of screening were classified as referred to tobacco treatment in error. ^c^Includes patients referred but not reachable by a tobacco treatment coordinator for scheduling and only received tobacco treatment educational material.

**Table.** Tobacco Use, Referral, and Referral Acceptance Status by Race and Ethnicity

Characteristic	No. of patients	Tobacco use status (n = 302 971)			Referral status (n = 18 475)^a^		Eligibility status (n = 16 094)	Reached status (n = 14 096)	Referral acceptance (n = 9767)^b^	
		Current, No. (%)	*P* value	Referred, No. (%)		*P* value	Eligible, No. (%)	Reached, No. (%)^c^	Accepted and scheduled, No. (%)	*P* value
Race										
Asian	20 742	788 (3.8)	<.001	682 (86.5)		.43	582 (85.3)	417 (71.6)	194 (46.5)	<.001
Black or African American	21 626	1545 (7.1)		1364 (88.3)			1185 (86.9)	870 (73.4)	577 (66.3)	
White	236 468	14 670 (6.2)		12 758 (87.0)			11 188 (87.7)	7692 (68.8)	4128 (53.7)	
Other^d^	24 135	1472 (6.1)		1290 (87.7)			1141 (88.4)	788 (69.1)	458 (58.1)	
Ethnicity										
Non- Hispanic or Latino/e/a/x	261 860	15 987 (6.1)	.02	13 948 (87.2)		.35	12 171 (87.3)	8439 (69.3)	4580 (54.3)	.005
Hispanic or Latino/e/a/x	21 515	1366 (6.3)		1182 (86.5)			1022 (86.5)	698 (68.3)	422 (60.5)	
Unknown or missing	19 596	1122 (5.7)		964 (85.9)			903 (93.7)	630 (69.8)	355 (56.3)	

^a^ Patients screened for tobacco use and self-reported currently using tobacco products.

^b^ Patients reached by a tobacco treatment coordinator and scheduled for an appointment with a tobacco treatment specialist.

^c^ Patients reached by a tobacco treatment coordinator and offered a counseling appointment.

^d^ Self-reported Alaska Native, American Indian, Hawaiian Native or Pacific Islander, multiracial, unknown, or refused to answer. These categories were derived from electronic health record.

## Discussion

These findings suggest that a standardized tobacco use assessment with opt-out referral to tobacco treatment in a large cancer care setting is feasible and achieves higher tobacco treatment referral and acceptance rates than typically observed.^2^ By normalizing tobacco use screening and treatment as essential elements of high-quality cancer care, a universal tobacco screening and opt-out referral strategy may mitigate the stigma associated with patient engagement in tobacco treatment.^3^ An opt-out referral model may eliminate clinician referral bias and thereby facilitate equitable access to and use of tobacco treatment services^1^ among racially and ethnically diverse patients with cancer. Our results are consistent with studies showing high tobacco treatment rates using an opt-out, population-based patient engagement method.^4,5,6^ Despite these encouraging findings, 12.8% of patients identified as currently using tobacco were not referred for treatment, and 30.7% could not be reached for scheduling, consistent with prior studies.^4,5^

Limitations include lack of adjustment for age, sex, cancer diagnosis, and other variables that may also be associated with patient engagement. Strong receptiveness of tobacco treatment in cancer care may not be generalizable to non–cancer care settings.

## Conclusions

These findings support that a universal screening and opt-out referral strategy may mitigate disparities in tobacco treatment access and use in cancer care. Our ongoing work will examine facilitators of health equity through qualitative interviews with Black, Hispanic, and Latino/e/a/x patients to better understand these findings.

## Notes

Supplement 1. **eFigure**. Memorial Sloan Kettering Cancer Center (MSKCC) Clinical Standard for Universal Tobacco Use Assessment and Opt-Out Tobacco Treatment Referral Workflow

Supplement 2.
Data Sharing Statement
