# Supplementary material for: Universal Tobacco Screening and Opt-Out Treatment Referral Strategy Among Patients Diagnosed With Cancer by Race and Ethnicity
Source: JAMA Netw Open. 2024 Apr 22;7(4):e249525. doi: 10.1001/jamanetworkopen.2024.9525 (PMC11036136; doi:10.1001/jamanetworkopen.2024.9525)
Supplement: Supplement 1. — eFigure. Memorial Sloan Kettering Cancer Center (MSKCC) Clinical Standard for Universal Tobacco Use Assessment and Opt-Out Tobacco Treatment Referral Workflow [file jamanetwopen-e249525-s001.pdf]

Supplemental Online Content

Bates-Pappas GE, Schofield E, Chichester L-AR, et al. Universal tobacco screening and opt-out treatment referral strategy among patients diagnosed with cancer by race and ethnicity. *JAMA Netw Open*. 2024;7(4):e249525. doi:10.1001/jamanetworkopen.2024.9525

eFigure. Memorial Sloan Kettering Cancer Center (MSKCC) Clinical Standard for Universal Tobacco Use Assessment and Opt-Out Tobacco Treatment Referral Workflow

This supplemental material has been provided by the authors to give readers additional information about their work.

© 2024 Bates-Pappas GE et al. *JAMA Network Open*.

eFigure. Memorial Sloan Kettering Cancer Center (MSKCC) Clinical Standard for Universal Tobacco Use Assessment and Opt-Out Tobacco Treatment Referral Workflow

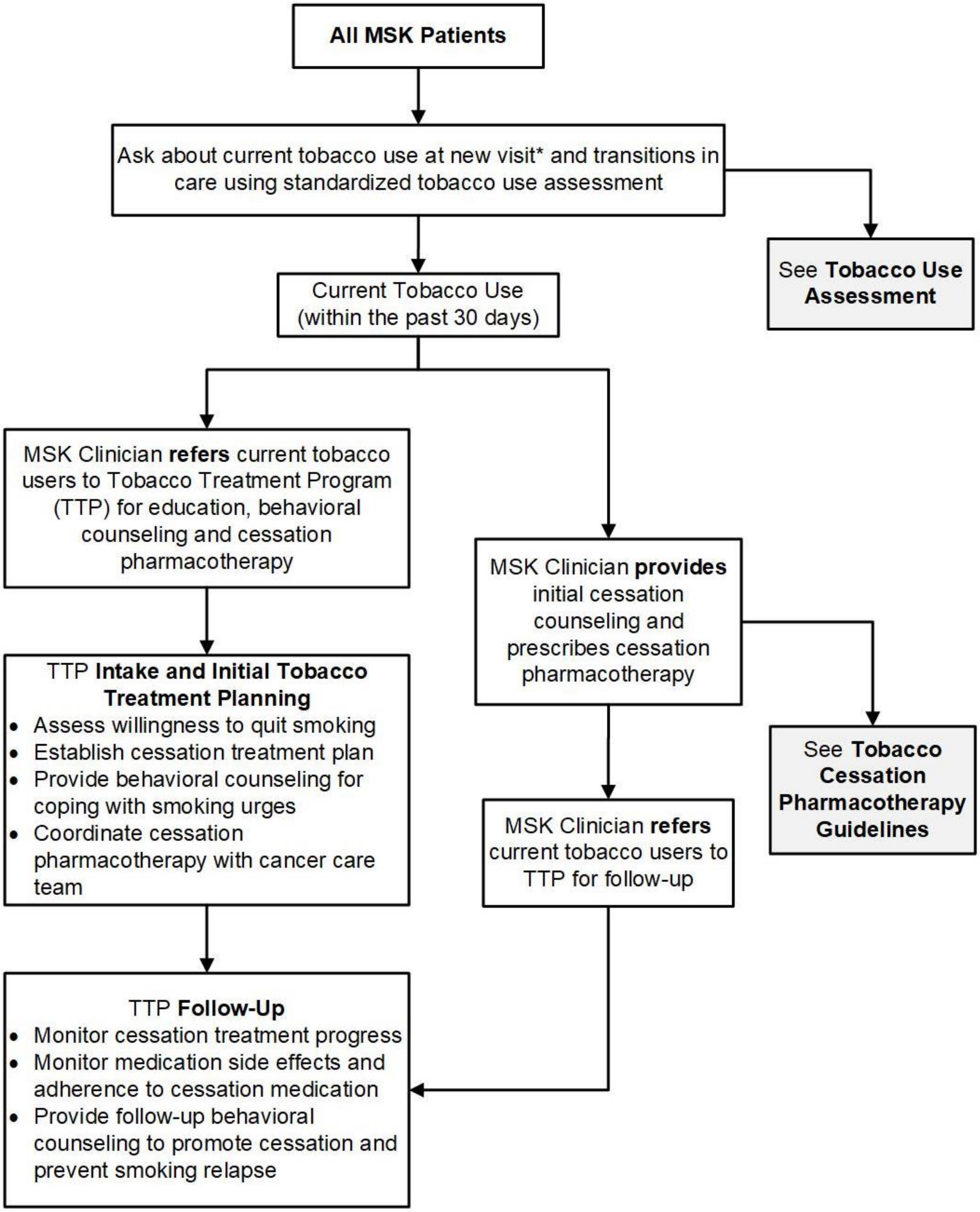

© 2024 Bates-Pappas GE et al. *JAMA Network Open*.
