# Supplementary material for: Universal Tobacco Screening and Opt-Out Treatment Referral Strategy Among Patients Diagnosed With Cancer by Race and Ethnicity
Source: JAMA Netw Open. 2024 Apr 22;7(4):e249525. doi: 10.1001/jamanetworkopen.2024.9525 (PMC11036136; doi:10.1001/jamanetworkopen.2024.9525)
Supplement: Supplement 2. — Data Sharing Statement [file jamanetwopen-e249525-s002.pdf]

Data Sharing Statement

Bates-Pappas. Universal Tobacco Screening and Opt-Out Treatment Referral Strategy Among Patients Diagnosed With Cancer by Race and Ethnicity. *JAMA Netw Open*. Published April 22, 2024. doi:10.1001/jamanetworkopen.2024.9525

Data
Data available: No
